# Development and calibration of a novel social relationship item bank to measure health-related quality of life (HRQoL) in Singapore

**DOI:** 10.1186/s12955-019-1150-9

**Published:** 2019-05-08

**Authors:** Yu Heng Kwan, Elenore Judy Uy, Dianne Carrol Bautista, Xiaohui Xin, Yunshan Xiao, Geok Ling Lee, Mythily Subramaniam, Janhavi Ajit Vaingankar, Mei Fen Chan, Nisha Kumar, Yin Bun Cheung, Terrance Siang Jin Chua, Julian Thumboo

**Affiliations:** 1 Program in Health Services and Systems Research, Duke-NUS Medical School, Singapore, Singapore; 2 Department of Rheumatology and Immunology, Singapore General Hospital, Academia Building, Level 4, 20 College Road, Singapore, 169856 Singapore; 3 Singapore Clinical Research Institute, Singapore, Singapore; 4 Centre for Quantitative Medicine, Duke-NUS Medical School, Singapore, Singapore; 5 Academic Clinical Programme for Medicine, Singapore General Hospital, Singapore, Singapore; 6 Department of Social Work, Faculty of Arts and Social Sciences, National University of Singapore, Singapore, Singapore; 7 Research Department, Institute of Mental Health, Singapore, Singapore; 8 Neuroscience and Mental Health, Lee Kong Chian School of Medicine, Singapore, Singapore; 9 Health Promotion Board, Singapore, Singapore; 10 Tampere Center for Child Health Research, University of Tampere and Tampere University Hospital, Tampere, Finland; 11 Department of Cardiology, National Heart Centre Singapore, Singapore, Singapore; 12 Office of Clinical, Academic and Faculty Affairs, Duke-NUS Medical School, Singapore, Singapore; 13 Yong Loo Lin School of Medicine, National University of Singapore, Singapore, Singapore

**Keywords:** Interpersonal relations, Quality of life, Psychometrics, Singapore, Culture, Asia

## Abstract

**Background:**

Social relationships (SR) is an important domain of health-related quality of life. We developed and calibrated a novel item bank to measure SR in Singapore, a multi-ethnic city in Southeast Asia.

**Methods:**

We developed an initial candidate pool of 51 items from focus groups, individual in-depth interviews and existing instruments that had been developed and/or validated for use in Singapore. We administered all items in English to a multi-stage sample of subjects, stratified for age and gender, with and without medical conditions, recruited from community and hospital settings. We calibrated their responses using Samejima’s Graded Response Model (SGRM). We evaluated a final 30-item bank with respect to Item Response Theory (IRT) model assumptions, model fit, differential item functioning (DIF), and concurrent and known-groups validity.

**Results:**

Among 503 participants (47.7% male, 41.4% above 50 years old, 34.0% Chinese, 33.6% Malay and 32.4% Indian), bi-factor model analyses supported essential unidimensionality: explained common variance of the general factor was 0.805 and omega hierarchical was 0.98. Local independence was deemed acceptable: the average absolute residual correlations were < 0.06 and 1.8% of the total item-pair residuals were flagged for local dependence. The overall SGRM model fit was adequate (*p* = 0.146). Five items exhibited DIF with respect to age, ethnicity and education, but were retained without modification of scores because they measured important aspects of SR. The SR scores correlated in the hypothesized direction with a self-reported measure of global health (Spearman’s rho = − 0.28, *p* < 0.001).

**Conclusion:**

The 30-item SR item bank has shown acceptable psychometric properties. Future studies to evaluate the validity of SR scores when items are administered adaptively are needed.

**Electronic supplementary material:**

The online version of this article (10.1186/s12955-019-1150-9) contains supplementary material, which is available to authorized users.

## Introduction

The World Health Organization (WHO) states that health is a state of complete physical, mental and social well-being, and not merely the absence of disease or infirmity. [1] SRs are defined as having deep and meaningful human connections – in other words, having good relationships with family, friends and others. [2, 3] SR is found to be an important determinant of health-related quality of life (HRQoL) in the literature. [4] Although there are static instruments such as Lubben Social Network Scale (LSNS) to measure SR, there is no item bank to measure SR in the adult population. [5]

There are item banks developed to measure social-related constructs such as social health before. [6, 7] One such example was an item bank that measured social health on an adult general population was developed on a very diverse latent construct that involved social role participation, social network quality, social integration and interpersonal communication. [6] This item bank may not be optimal to meaningful measure social relationship. Being able to measure how deep and meaningful an individual’s social relationships are, will facilitate interventions to be developed or refined to improve SR. [8]

To address the gap, we developed a comprehensive and culturally sensitive SR item bank to measure SR in Singapore. The aim of this study was to calibrate an item bank of SR that includes important and culturally appropriate items measuring SR that can be used across different age, gender and ethnic groups. A successfully calibrated item bank will allow us to develop CAT or short static instruments to measure SR in Singapore, whose multi-ethnic, English speaking population is in some ways a microcosm of Asia.

## Methods

This institutional board review-approved study (Ref 2014/916/A) consisted of the following sequential steps: development of a candidate item bank, administration of this candidate item bank via a community and hospital-based survey, and item bank calibration through assessing the assumptions of item response theory (IRT), fitting the responses to an IRT model, testing for differential item functioning (DIF) and testing the SR scores of the item bank using a priori hypotheses. In this manuscript, we will describe the details of the SR item bank calibration. The development of the calibrated item bank has been separately described and is briefly summarised below. [3, 9, 10]

### Development of a candidate item bank

Methodological details for developing candidate items had been reported separately. [3, 9, 10] In brief, we adapted the PROMIS Qualitative Item Review (QIR) protocol [11], with input and endorsement from expert panels (comprising patients, members of the general public, and experts in psychology, social work and psychometrics). Items were generated from thematic analyses from focus groups and in-depth interviews and a literature search to identify studies that developed or validated a health-related quality of life instrument among adults in Singapore. Items from these sources were “binned” and “winnowed” (as detailed in the PROMIS QIR protocol) by two independent reviewers, blinded to the source of the items, who harmonized their selections to generate a list of candidate items (each item representing a sub domain). An expert panel reviewed and refined the face and content validity of these candidate items.

### A community and hospital-based survey

We recruited English and Mandarin speaking Singapore citizens or permanent residents from the community and from the specialist outpatient clinics of Singapore General Hospital and National Heart Centre Singapore to sample subjects with and without illnesses, who would be expected to have a wider spectrum of social relationships. Within each language sampling frame, a purposive sample of participants was drawn based on age, gender, ethnicity and presence or absence of chronic illnesses. The list of chronic illnesses was based on the Singapore Burden of Disease Study [12] and is detailed in Additional file 1: Table S1. The presence or absence of a chronic illness was based on a participant’s self-report of having been diagnosed of an illness by a physician. Participants were categorized into well, mildly unwell, and unwell, according to the number and severity of chronic illnesses. We excluded individuals who had impairments that precluded a meaningful exchange of ideas or other conditions that prohibited them from carrying out a normal interview, such as severe mental illness and cognitive impairment. In order to include participants with a wide spectrum of health, we predefined the proportion of participant recruitment in health categories to be 35% well, 15% mildly unwell, and 50% unwell.

**Fig. 1:**
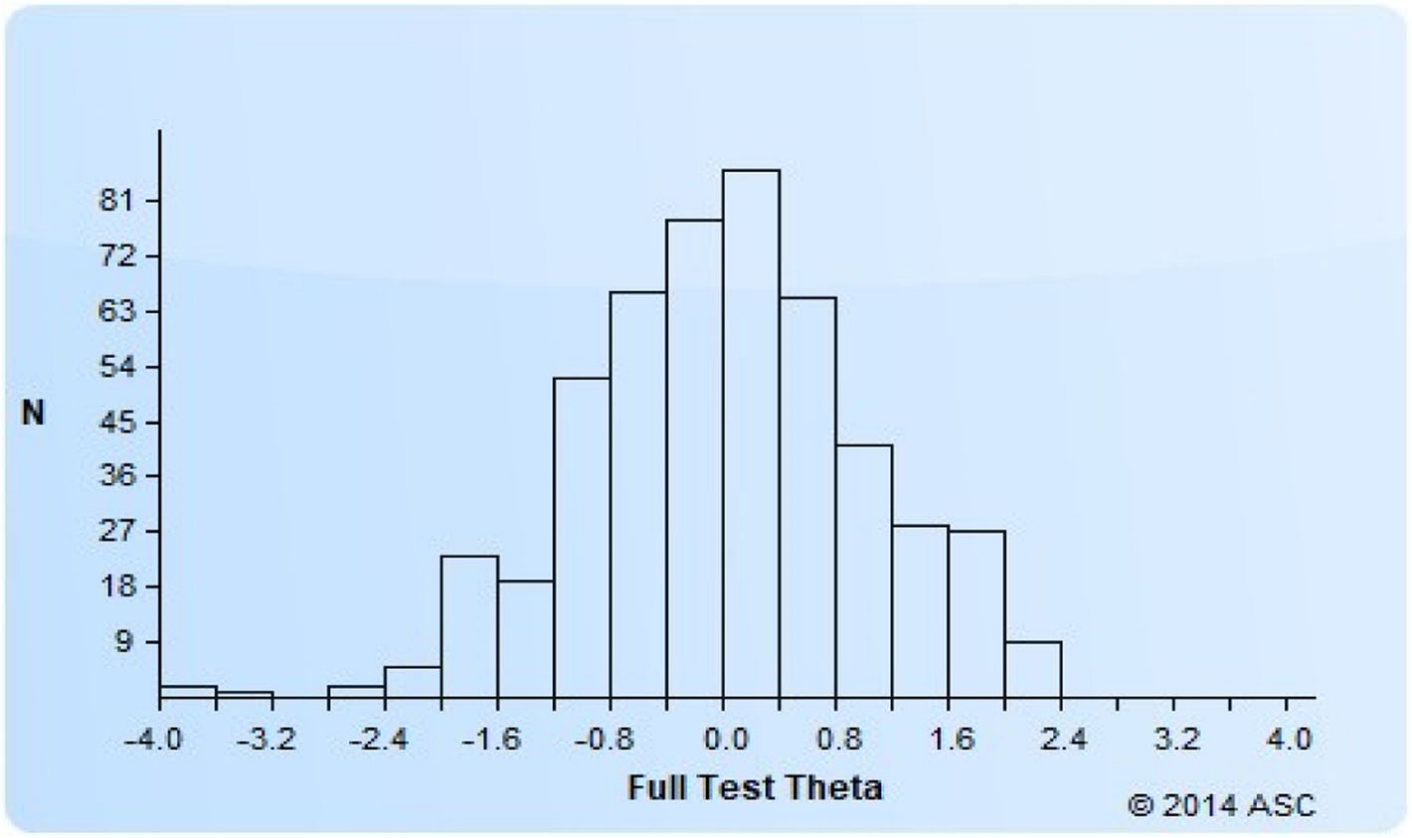
The theta range for the SR item bank

Participants from the community were sampled using a proprietary sampling frame of public housing which accounts for 82% of Singapore residential households [13]. The primary sampling units were plots of land with approximately equal numbers of households, stratified according to geographic location and dwelling type. Households in each primary sampling unit were selected based on fixed route rules and skip patterns based on pre-specified ethnic and age quotas. Only one respondent per household was selected for a face-to-face interview. Three call attempts to each household were made at different times of the day with at least 1 visit on a non-work day (Saturday or Sunday). This residential-household-based sampling method has been used in the Singapore National Health Survey since 2004 [14, 15]. The response rate of the study was computed using the standard set by the Council of American Survey Research Organization [16], generally defined as the number of completed interviews divided by the number of eligible reporting units in sample. We engaged a research company to conduct the standardized surveys on behalf of the study team.

We recruited participants to test the response for all 3 of our item banks (Physical Function, Positive Mindset and Social Relationship). Each recruited participant was administered the items for only one of the three domains, in either English or Mandarin. The survey was administered by trained interviewers. We chose to have the survey as interviewer-administered rather than self-administered so that illiterate subjects (who form 20% of Singapore population) could be included and the resulting item bank could be applied to all English and Mandarin speakers in Singapore. [17] There were 51 candidate items presented to the participants with 5-level item response options adapted from the PROMIS. The response options were “Never”, “Seldom”, “Sometimes”, “Usually” and “Always” for items on frequency and “Not at all”, “Mildly”, “Moderately”, “Quite a lot” and “Extremely” for items on intensity. We collected demographic information including age, gender, ethnicity, education, and current marital status. We collected a single-item, participant-reported assessment of global health for comparison.

### Item bank calibration

We adapted the methodology published by PROMIS to calibrate the SR item bank. To assess Item Response Theory (IRT) model assumptions, we performed the following: for unidimensionality, we used factor analyses, which involved Exploratory (EFA) and Confirmatory (CFA) and Exploratory bifactor analyses with orthogonal rotation. If EFA and CFA indicated secondary dimensions, we provided details of the latter. In the bifactor analyses, we used (1) the average relative parameter bias (ARPB) which is the mean of the absolute differences between item loadings on the unidimensional model and item loadings in the bifactor’s general factor [18], (2) the explained common variance (ECV) of the general factor, (3) omega hierarchical (omegaH) and (4) item ECVs (IECVs) to judge whether manifestations of secondary dimensions do not bar the instrument’s interpretation of the construct as being predominantly unidimensional. For local independence, we examined the residual correlation matrix from the single factor CFA and where applicable, the residual correlation matrix from bifactor analyses as well. We state the criteria and thresholds for appraising IRT model assumptions in Table 2. We used Mplus Version 8.0 software to verify unidimensionality and local independence [19]. We adopted Samejima’s graded response model (GRM) and estimated parameters via marginal maximum likelihood using the Xcalibre 4.2 IRT software (Assessment Systems Corporation, USA). We checked the adequacy of the overall model fit and individual item fits using a chi-square-based fit statistic. We examined differential item functioning (DIF) by these subgroups: age (age < 50 versus age ≥ 50), gender (Male/Female), ethnicity (Chinese vs non-Chinese) and education (completers of secondary education vs non-completers), by means of likelihood chi-square statistics from nested ordinal logistic regression models, assessing the incremental contribution of subgroup membership at a 5% level of significance. We assessed both uniform and non-uniform DIF using a specially written syntax for IBM Statistics Version 23.0 (http://www-01.ibm.com/support/docview.wss?uid=swg21572191, downloaded on 18 December 2017). We evaluated the 30 SR items for concurrent validity using a self-reported measure of global health (1 = Excellent health, 2 = Very good, 3 = Good, 4 = Fair, 5 = Poor), positing a moderate negative correlation (Spearman’s rho < − 0.25) between SR theta scores and the global health self-report. We also verified that adjusted means of global health categories showed a decreasing trend. Adjustment was made for participant’s age (20–35, 36–49, 50 and above), gender (Male/Female), completion of secondary education (Yes/No) and current marital status (Single, Married, Divorced/Widowed/Separated). We used a 5% significance level. Evaluations of concurrent validity were implemented in IBM Statistics Version 25.0 software.

## Results

Of 8027 contacted subjects, 4918 were eligible (see Additional file 1: Figure S1 for details). We implemented a quota system for eligible subjects, as a result of which 41.2% (2034/4918) of eligible subjects were surveyed, while 2851 eligible subjects were excluded as their quotas had been met. All set quotas for sociodemographic categories were achieved within 5% of differences. Thus a total of 2034 Singapore citizens or permanent residents (consisting of 1170 subjects from hospital-based specialist outpatient and 864 subjects from the community) completed one of 3 item banks (SR, physical functioning, and positive mindset), of which 679 subjects completed the SR item bank survey in English (*n* = 503) or Chinese (*n* = 173). This paper focuses on the analysis and calibration of the English SR item bank. Characteristics of the study participants are shown in Table 1. The full range of theta of the SR item bank is presented in Fig. 1.

**Table 1:** Characteristics of study subjects

	Frequency (%)
	*N* = 503
Age	
21–34 years old	122 (24.3)
35–49 years old	173 (34.4)
50 and above	208 (41.4)
Gender	
Male	240 (47.7)
Female	263 (52.3)
Ethnic Group	
Chinese	171 (34.0)
Malay	169 (33.6)
Indian	163 (32.4)
Health^1^	
Well	180 (36.5)
Mildly unwell	62 (12.6)
Unwell	251 (50.9)
Marital status	
Single	110 (21.9)
Married	364 (72.4)
Separated/divorced/widowed	29 (5.8)
Completion of secondary education (10 years of education)	
Yes	422 (83.9)
No	81 (16.1)

^1^Based on the list of chronic diseases as defined in Additional file 1: Table S1

### Item analyses

Thirty of 51 candidate items were retained in the final SR item bank after reviewing initial IRT model fits and adequacy checks and consulting with the expert panel. The 30 items showed a very high inter-item consistency with a Cronbach’s alpha of 0.96. Item means varied from 2.76 to 4.58 with a mean of 4.24 and standard deviation of 0.36. The mean item-to-total score correlation was 0.65 (SD = 0.12). The percentage of non-response did not exceed 0.2%.

### IRT assumptions of unidimensionality and local independence

Unidimensionality was evaluated with EFA, CFA and bifactor analyses. In the EFA, the first factor accounted for 18.1% of the variance and the ratio of the first and second highest eigen values was 8.01 (Table 2). In the CFA, the results indicated the presence of secondary dimensions based on Comparative Fit Index (CFI) < 0.95, Tucker-Lewis Index (TLI) < 0.95 and Root Mean Square Error of Approximation (RMSEA) > 0.06 (Table 2). In the light of EFA and CFA results, we pursued exploratory bifactor analyses specified with two, three and four specific factors. The results showed that the presence of secondary dimensions did not impede the interpretation of the item bank as being predominantly unidimensional: the ARPB < 10% [18]*,* the minimum ECV and omegaH values were respectively 0.80 and 0.98 which are much higher than Reise et al’s suggested criteria (ECV > 0.60 and omegaH> 0.70) [20]. Therefore, the item bank can be regarded as being essentially unidimensional. This interpretation was reinforced by mean item ECVs which were mostly above 0.80 (Table 3). Inspection of the single-factor CFA residual correlation matrix revealed little local dependence: the mean of the residual correlations was < 0.07 which was less than the 0.1 threshold. The proportion of item-pairs having problematic residual correlations (i.e., greater than 0.20) was 1.8% (8 of 435). Items 1 (“*I have a good relationship with my family*”) and 16 (“*I keep in touch with my friends*”) accounted for 4 out of the 8 problematic residual correlations. Examination of the bifactor residual correlation matrices (across models with two, three and four specific factors) showed a maximum mean residual correlation was 0.026 which is less than the threshold of 0.10. In all three bifactor models, the percentage of problematic residual correlations was < 0.1%. We thus judged the degree of local dependency to be slight as not to bias the accuracy of IRT parameter estimation.

**Table 2:** Criteria for evaluating adequacy of meeting IRT assumptions and results

Unidimensionality				
Approach	Criterion	Reference	Results	Criterion met?
EFA	Percentage of variance accounted for by first factor > 20%	PROMIS [16]	18.1%	No
	Ratio of first to second eigenvalues > 4.0	PROMIS [16]	8.01	Yes
CFA	CFI > 0.95	PROMIS [16]	0.923	No
	TLI > 0.95	PROMIS [16]	0.917	No
	RMSEA < 0.06	PROMIS [16]	0.098	No
	SRMR < 0.08	PROMIS [16]	0.081	No
Bifactor analyses	ARPB < 10%	Muthén, Kaplan, and Hollis (1987) [33]	3.6%^§^	Yes
	General ECV > 0.70	Reise, Bonifay and Haviland (2013) [17]	0.805^ǂ^	Yes
	OmegaH > 0.80		0.980^¥^	Yes
	General ECV > 0.60 and OmegaH> 0.70	Reise, Schienes, Widaman and Haviland (2013) [15]		Yes
Local Independence				
Residual correlation matrix	Average absolute residual correlations < 0.10	PROMIS[16]	0.067	Yes
	Percentage of residual correlations above 0.20	Artmann et al. 2010 [18]	1.84%	No threshold given
			(8 of 435)	

Abbreviations: Item response theory (IRT), comparative fit index (CFI), Tucker-Lewis Index (TLI), root mean square error of approximation (RMSEA), standardized root mean residual (SRMR), explained common variance (ECV), item explained common variance (IECV), omega hierachical (omegaH), explanatory factor analysis (EFA), confirmatory factor analysis (CFA)

^§^Maximum ARPB among three exploratory bifactor models with 2,3 and 4 specific factors. See Table 3

^ǂ^Minimum general factor ECV attained among three exploratory bifactor models with 2, 3 and 4 specific factors. See Table 3

^¥^ Minimum OmegaH attained among three exploratory bifactor models. See Table 3

**Table 3:** Summary of results of bifactor analyses

Item ID	Item	Single-factor CFA	Exploratory Bifactor Analysis – general factor loadings			
			2 specific factors	3 specific factors	4 specific factors	Mean Item ECVs^§^
SQ01	I have a good relationship with my family	0.931	0.900	0.895	0.908	0.884
SQ32	I have someone to talk to about my problems	0.792	0.803	0.796	0.801	0.952
SQ39	I live harmoniously with others	0.790	0.800	0.796	0.798	0.941
SQ11	I communicate well with my family	0.940	0.921	0.915	0.926	0.918
SQ02	I participate in family activities	0.757	0.769	0.765	0.769	0.952
SQ33	I have someone who can provide me with information if I need it	0.777	0.789	0.785	0.786	0.974
SQ10	I take care of my family	0.772	0.768	0.775	0.782	0.835
SQ40	I communicate well with others	0.841	0.843	0.847	0.844	0.847
SQ12	I keep in touch with my family	0.879	0.864	0.870	0.878	0.897
SQ07	My family is willing to help with my daily tasks (e.g. shopping, giving me a ride, or helping me with household tasks) when I need it	0.700	0.711	0.709	0.712	0.922
SQ16	I keep in touch with my friends	0.762	0.689	0.699	0.671	0.598
SQ34	I know that I have someone to help me if I have financial difficulties	0.680	0.667	0.661	0.652	0.826
SQ41	I keep in touch with others	0.812	0.780	0.787	0.768	0.788
SQ04	I feel loved and cared for by my family	0.858	0.849	0.855	0.866	0.900
SQ08	My family is willing to give me information when I need it	0.835	0.828	0.831	0.841	0.895
SQ17	I communicate well with my friends	0.766	0.756	0.770	0.754	0.844
SQ43	I am able to socialize with others	0.718	0.702	0.717	0.701	0.798
SQ45	I feel loved and cared for	0.852	0.863	0.871	0.872	0.971
SQ31	I have someone I can go to for advice if I need it	0.763	0.757	0.744	0.749	0.703
SQ35	I give help to others	0.712	0.700	0.703	0.693	0.813
SQ44	I have someone who would spend time with me when I need company	0.827	0.822	0.815	0.811	0.858
SQ05	Overall, my family supports me when I need it	0.835	0.826	0.820	0.833	0.858
SQ18	I have gatherings with my circle of friends	0.686	0.630	0.628	0.606	0.607
SQ15	I give support to my friends	0.674	0.647	0.651	0.635	0.771
SQ37	I get along well with others	0.750	0.759	0.763	0.761	0.967
SQ06	My family is willing to listen when I need to talk about my worries and problems	0.788	0.783	0.774	0.788	0.830
SQ20	I spend time with my friends	0.670	0.598	0.600	0.576	0.536
SQ48	Overall, I am satisfied with the support I get from my friends	0.741	0.715	0.695	0.680	0.712
SQ50	Overall, I am satisfied with the support I give to others	0.753	0.732	0.705	0.694	0.662
SQ51	Overall, I am satisfied with how well I communicate with others	0.797	0.793	0.770	0.767	0.806
Bifactor analysis statistics						
ARPB			2.6	2.8	3.6	
ECV			0.855	0.828	0.805	
OmegaH			0.983	0.985	0.984	
Hancock and Mueller’s H			0.995	0.992	0.985	
Average ECVs of specific factors			0.072	0.057	0.049	

Data within single factor CFA, bifactor analysis (2-specific factors, 3-specicifc factors and 4-specific factors) represented factor loadings. Abbreviations: Confirmatory factor analysis (CFA), explained common variance (ECV), omega hierarchical (omegaH)

### IRT calibration and fit

SR items were summed so that higher scores reflected better social relationships. The overall fit of the GRM was found to be adequate (chi-square = 1710.53, df = 1650, *p* = 0.146). The items and parameter estimates are given in Table 4. Setting the level of significance at 0.01 for GRM item fit, the model did not fit well for three items: Items 34 (“*I know that I have someone to help me when I have financial difficulties.*”), 20 (*“I spend time with my friends.”*) and 50 (*“Overall, I am satisfied with the support I give to others.”*). For all other items, *p* values ranged from 0.03 to 1.00 with a mean of 0.55. The median of item discrimination parameters was 1.22 (mean = 1.24, median = 1.43).

**Table 4:** IRT Calibration Results of SR item bank

Item ID	Mean	Item-theta correlation	Cronbach’s alpha if deleted	IRT					GRM item fit *p*-value
				Discrimination	B1	B2	B3	B4	
SQ01	4.584	0.711	0.961	2.072	−2.43	−2.18	−1.75	0.55	1.000
SQ32	4.35	0.713	0.961	1.509	−2.74	− 2.25	− 1.39	−0.09	0.734
SQ39	4.531	0.673	0.962	1.378	−3.54	−3.18	− 2.06	− 0.30	0.193
SQ11	4.546	0.754	0.961	2.081	−2.58	− 2.29	−1.71	− 0.42	1.000
SQ02	4.175	0.672	0.962	1.179	−2.63	−1.97	− 1.13	0.06	0.711
SQ33	4.286	0.71	0.961	1.266	−2.94	− 2.40	−1.33	0.07	0.714
SQ10	4.493	0.629	0.962	1.191	−2.76	− 2.29	−1.81	− 0.46	0.086
SQ40	4.483	0.713	0.961	1.418	−4.16	−3.00	−1.74	−0.23	0.997
SQ12	4.553	0.702	0.961	1.608	−2.77	−2.30	−1.65	−0.53	0.981
SQ07	4.171	0.641	0.962	1.021	−2.89	− 2.09	−1.17	0.12	0.446
SQ16	4.125	0.627	0.962	0.963	−3.5	−2.36	−1.08	0.29	0.226
SQ34	3.917	0.607	0.962	0.95	−2.33	−1.68	−0.79	0.29	0.000
SQ41	4.235	0.692	0.961	1.23	−3.16	−2.24	−1.13	0.04	0.030
SQ04	4.545	0.692	0.961	1.54	−2.64	− 2.28	−1.65	−0.53	0.888
SQ08	4.453	0.711	0.961	1.435	−2.78	−2.29	−1.55	−0.30	0.997
SQ17	4.376	0.66	0.962	1.153	−3.77	−2.88	−1.50	−0.11	0.613
SQ43	4.326	0.607	0.962	1.007	−3.95	−2.74	−1.47	−0.08	0.229
SQ45	4.517	0.72	0.961	1.634	−2.83	−2.41	−1.63	−0.38	0.828
SQ31	4.266	0.675	0.961	1.229	−2.73	−2.32	−1.32	0.02	0.630
SQ35	4.26	0.642	0.961	1.044	−3.42	−2.57	−1.29	0.06	0.402
SQ44	4.292	0.734	0.961	1.464	−3.24	−1.97	− 1.22	−0.04	0.060
SQ05	4.503	0.674	0.961	1.445	−2.5	−2.36	−1.58	−0.48	0.546
SQ18	3.817	0.6	0.962	0.863	−2.96	−1.75	−0.62	0.64	0.073
SQ15	4.024	0.586	0.962	0.896	−3.01	−2.56	−1.04	0.54	0.191
SQ37	4.475	0.66	0.962	1.221	−4.35	−3.36	−1.89	−0.18	0.949
SQ06	4.345	0.673	0.961	1.256	−2.56	−2.19	−1.41	−0.18	0.999
SQ20	3.787	0.575	0.962	0.794	−3.48	−1.97	−0.51	0.80	0.005
SQ48	4.167	0.663	0.962	1.138	−2.75	−2.11	−1.14	0.13	0.078
SQ50	2.765	0.074	0.969	0.157	−3.95	−0.11	2.26	5.29	0.000
SQ51	3.974	0.656	0.962	1.05	−2.73	−2.20	−1.01	0.73	0.321

Abbreviations: Social relationships (SR), item response theory (IRT), graded response model (GRM)

### Differential item function detection

At the 1% level of significance, none of the items had gender-related DIF but five items were found to have significant DIF in age, ethnicity and education. The two items with non-uniform age-related DIF were Items 10 (*“I take care of my family.”*) and 51 (*“Overall, I am satisfied with how well I communicate with others.”*). In ethnicity, Items 18 (*“I have gatherings with my circle of friends.”*) and 8 (*“My family is willing to give me information when I need it.”*) were respectively found to have uniform and non-uniform DIF. In education, both Items 10 and 16 (*“I keep in touch with my circle of friends.”*) displayed non-uniform DIF.

### Concurrent validity evaluation

The spearman correlation between SR scores and self-reported global health was r = − 0.28 (95% CI: -0.359 to − 0.196), supporting the hypothesis of a moderate correlation between the two measures. After accounting for age, gender, completion of secondary education and current marital status, the adjusted means of the ordered categories of global health likewise showed a decreasing trend (Table 5). Both these findings supported the concurrent validity of the SR item bank.

**Table 5:** Evaluation of concurrent validity

Self-report of global health	*N* (%)	SR theta score means^#^ (95% CI)	Spearman’s rho
			(95% CI)
Excellent	32 (6.4)	0.29 (−0.12 to 0.70)	−0.279
Very Good	119 (23.7)	- 0.05 (−0.33 to 0.23)	(−0.359, − 0.196)
Good	255 (50.7)	- 0.26 (−0.51 to − 0.02)	
Fair	77 (15.3)	−0.71 (−1.02 to − 0.39)	
Poor	20 (4.0)	−1.32 (− 1.78 to − 0.86)	

^#^adjusted for sex (male vs female), age group (21–34, 35–49, 50 and above), ethnicity

(Chinese, Malay, Indian), completion of secondary education (Yes vs No) and current marital status

(Married vs Single/Separated/Divorced)

Abbreviations: Social relationships (SR), confidence interval (CI)

## Discussion

This study describes the calibration of a culturally sensitive item bank for SR. Items from this SR item bank were derived from (1) qualitative research to identify and incorporate perspectives from subjects in the population, representing a wide spectrum of healthy and ill subjects (with chronic diseases) and (2) Items from developed static instruments measuring related concepts in the same population. The item bank we developed thus has high content validity. The calibration processes aligned with the approach espoused by the PROMIS group [20–25]. The findings of this successful calibration indicate that this social relationship item bank is a promising tool for measuring SR.

The analyses of the IRT assumptions show that the assumptions of essential unidimensionality and local independence are met. The bifactor model results exceeded the recommended thresholds. [21] DIF tests for age, ethnicity and education identified five items – however the impact of DIF was modest. In item bank development, statistical methods were used to inform, and not to decide item selection. [26] Therefore, items were retained because of their importance and the modest impact of DIF [27, 28].

SR is a novel construct which has wide-ranging impact on health and its measurement is thus important to improve health. For example, high SR has been shown to improve social support and ameliorate the impact of diseases on overall health. [29] High SR has also been shown to be associated with low mortality, improved immune function and also delay the development of cardiovascular disease. [30] Given this, the SR item bank has several potential uses – for example as an outcome measure for individual- or family-based cohort studies or interventional trials in community or hospital-based settings. [31]

This study also supports the concurrent construct validity of the SR item bank. Our hypothesis testing showed moderate correlation between the SR scores and self-reported global health. Good social relationship may contribute to better health status due to stronger social support. [32] Another possible use of the SR item bank may therefore be to screen for people with poor social support and intervene as appropriate. However, further studies are needed to validate the SR item bank as a screening tool.

We recognize several limitations of this study. First, a significant number of eligible subjects were excluded because the quota for these subjects had been met. However, partly because of the use of quota sampling, the demographics in our sample are comparable to that of the population in Singapore. [33] Second, the SR item bank may have poorer coverage on higher SR trait but better coverage on lower SR trait. The SR item bank will be most useful to identify people at risk of impaired social relationship or people who are in need of social support. [32]

## Conclusions

We developed and calibrated a 30-item bank for SR that is relevant to the Singaporean population and applicable to healthy adults and those having chronic illnesses. This item bank shows promise and will subsequently be used to develop relevant short-form tests or CATs to facilitate routine clinical use.

## Additional file

Additional file 1: **Table S1.** Chronic Illnesses qualifying for patient recruitment. **Figure S1** Flow chart describing the response rate. (DOCX 35 kb)

## Abbreviations

ANOVA: Analysis of Variance
CAT: Computerised Adaptive Testing CFA Confirmatory factor analysis
CFI: Comparative Fit Index
DIF: Differential Item Functioning
ECV: Explained Common Variance
EFA: Explanatory Factor Analysis
GRM: Graded Response Model
HRQoL: Health-Related Quality of Life
IECV: Item Explained Common Variance
IRT: Item Response Theory
OmegaH: Omega Hierarchical
PROMIS: Patient Reported Outcome Measurement Information System
QIR: Qualitative Item Review
RMSEA: Root Mean Square Error of Approximation
SF-36: Short Form-36
SR: Social Relationship
TLI: Tucker Lewis Index
WHO: World Health Organization
WHOQOL-BREF: World Health Organization Quality of Life Scale
