# Supplementary material for: Development and calibration of a novel social relationship item bank to measure health-related quality of life (HRQoL) in Singapore
Source: Health Qual Life Outcomes. 2019 May 8;17:82. doi: 10.1186/s12955-019-1150-9 (PMC6505203; doi:10.1186/s12955-019-1150-9)
Supplement: Supplementary file 1 — Table S1. Chronic Illnesses qualifying for patient recruitment. Figure S1 Flow chart describing the response rate. (DOCX 35 kb) [file 12955_2019_1150_MOESM1_ESM.docx]

**Supplementary Table 1.** Chronic Illnesses qualifying for patient recruitment

| **List A** | **List B** |
| --- | --- |
| - Age-related macular degeneration - Anxiety disorder - Asthma - Breast cancer - Chronic obstructive pulmonary disease (COPD) - Colon cancer - Depression - Diabetes - Diabetic retinopathy - Glaucoma - Hearing loss, adult-onset - Heart failure - Ischemic heart disease - Lung cancer - Migraine - Osteoarthritis - Rheumatoid arthritis - Stroke | - Cancer except breast or color cancer - Epilepsy - Heart disease except heart failure, ischemic heart disease - High cholesterol - Hypertension - Joint disease except rheumatoid arthritis or osteoarthritis - Kidney disease not requiring dialysis or transplantation - Benign prostate hyperplasia - Osteoporosis |

Based on the 2010 Singapore Burden of Disease Study ^13^

**No contact made: 9,107 (C: 8,968, H: 139)**

No reply at when called or at door after 3 attempts: 8930 (C: 8,930, H: 0)

Vacant or demolished unit: 38 (C: 38, H: 0)

Did not acknowledge interviewer’s presence: 139 (C:0, H: 139)

Sampling frame: 17,134

(C: 14,324, H: 2,810)

Contacted: 8,027

(C: 5,356, H: 2,671)

**Not eligible: 3,109 (C: 2,083, H: 1,026)**

Did not speak English or Chinese: 126 (C: 50, H: 76)

Declined to participate during screening: 2137 (C: 1,512, H: 625)

Below 21 or above 90 years old: 170 (C: 121, H: 49)

Not Singapore citizen or permanent residents: 209 (C: 162, H: 47)

Not Chinese, Malay or Indian: 98 (C: 65, H: 33)

Cognitive impairment: 98 (C: 65, H: 33)

Did not fulfill chronic disease criteria: 149 (C: 29, H: 120)

Participated in a survey in the past month: 53 (C: 0, H: 53)

Eligible: 4,918

(C: 3,273, hospital: 1,645)

Total interviewed: 2,034

(C: 864, hospital: 1,170)

**Eligible but not interviewed: 2,884 (C: 2409, H: 475)**

Gender quota filled: 237 (C: 203, H: 34)

Age quota filled: 1,210 (C: 1,128, H: 82)

Ethnicity quota filled: 609 (C: 573, H: 36)

Chronic disease status quota filled: 493 (C: 241, H: 252)

Language quota filled: 104 (C: 79, H: 25)

Survey version quota filed: 198 (C: 169, H: 29)

Declined to give informed consent: 9 (C: 0, H: 9)

Declined to give personal details: 15 (C: 8, H: 7)

Declined to continue: 9 (C: 8, H: 1)

**Supplementary Figure 1.** Flow chart describing the response rate

Abbreviations: community (C); hospital (H)
